# Southeast Asian protected areas are effective in conserving forest cover and forest carbon stocks compared to unprotected areas

**DOI:** 10.1038/s41598-021-03188-w

**Published:** 2021-12-09

**Authors:** Victoria Graham, Jonas Geldmann, Vanessa M. Adams, Pablo Jose Negret, Pablo Sinovas, Hsing-Chung Chang

**Affiliations:** 1 Department of Earth and Environmental Sciences, Macquarie University, Sydney, Australia; 2 Center for Macroecology, Evolution and Climate, Globe Institute, University of Copenhagen, Copenhagen, Denmark; 3 Conservation Science Group, Department of Zoology, University of Cambridge, Downing St, Cambridge, CB2 3EJ UK; 4 School of Geography, Planning, and Spatial Sciences, University of Tasmania, Hobart, Australia; 5 Department of Biological Sciences, Macquarie University, Sydney, Australia; 6 Centre for Biodiversity and Conservation Science, University of Queensland, Brisbane, Australia; 7 Fauna and Flora International, Phnom Penh, Cambodia

**Keywords:** Environmental impact, Conservation biology, Ecosystem services, Tropical ecology

## Abstract

Protected areas aim to conserve nature, ecosystem services, and cultural values; however, they have variable success in doing so under high development pressure. Southeast Asian protected areas faced the highest level of human pressure at the turn of the twenty-first century. To estimate their effectiveness in conserving forest cover and forest carbon stocks for 2000–2018, we used statistical matching methods to control for the non-random location of protected areas, to compare protection against a matched counterfactual. We found Southeast Asian protected areas had three times less forest cover loss than similar landscapes without protection. Protected areas that had completed management reporting using the Management Effectiveness Tracking Tool (METT) conserved significantly more forest cover and forest carbon stocks than those that had not. Management scores were positively associated with the level of carbon emissions avoided, but not the level of forest cover loss avoided. Our study is the first to find that METT scores could predict the level of carbon emissions avoided in protected areas. Given that only 11% of protected areas in Southeast Asia had completed METT surveys, our results illustrate the need to scale-up protected area management effectiveness reporting programs to improve their effectiveness for conserving forests, and for storing and sequestering carbon.

## Introduction

Natural forests have high conservation value in terms of irreplaceable habitat for a disproportionately large number of species as well as for the ecosystem services they provide, such as carbon sequestration and storage^1,2^. Populations of vertebrates have declined by 68% on average between 1970 and 2016^3^ and the extent of ‘healthy’ forests has shrunk to 40% of pre-human extent^4^. Some scientists warn that forests have gone beyond the precautionary ‘safe limit’ for land-system change as the level of forest cover loss has disturbed the biogeophysical processes of earth systems that directly regulate climate^5^. Protecting forests from intense levels of human pressure is essential for stemming the accelerating loss of species and their habitats, and for maintaining ecosystem services^6^. The last three decades have seen a considerable expansion of terrestrial and marine protected areas, which coincides with targets agreed under the Convention on Biological Diversity, effective from 1993. However, expanding the coverage of protected areas alone has proved insufficient to protect biodiversity; effective management within protected area borders is an essential component of ensuring species’ populations are stable or improving^7^.

Rigorous evaluations of protected area performance remain relatively sparse, especially in the tropical zone^8^. Evidence depicting why some protected areas perform well, while others do not, is even scarcer^9^, yet this knowledge is crucial to improve the effectiveness of the protected area network. Further, many previous studies fail to account for the non-random location of protected areas by comparing areas that are protected to all the unprotected areas present in the landscape^10^, or to neighbouring buffers^11^. These approaches ignore any bias in protected area placement towards locations that are remote (i.e., high altitude, steep, far from urban centres or roads) and have less potential for agricultural production, which in turn affects the level of deforestation pressure^11–13^. Changes in forest cover or human pressure are two widely used indicators to measure the level of exposure to threats^14–16^. The advantage of such indicators is their broad coverage, that increases the comparability across sites. Separating the impact of law enforcement and management from protected area location can be achieved by comparing protected areas to counterfactual sites with similar characteristics. When using a counterfactual approach, estimates of the impact of protection on avoiding deforestation were consistently reduced^17–19^.

Based on Landsat satellite imagery between 2000–2013, it is estimated the global protected area network lost 3.4 times less intact forest than unprotected areas, accounting for differences in accessibility, terrain and human pressure^20^. Geographically, protection had the weakest effect on maintaining intact forests in Southeast Asia, Australia and tropical South America^20^. Indo-Malayan forests have exceptional biological diversity, yet also rapid rates of deforestation^21^, driven by global demand for timber, palm oil and rubber^22^. Protected areas in Southeast Asia experienced a higher mean change in human pressure between 1995 and 2010 than in any other region in the world^14^. Given historical deforestation rates, Sodhi et al.^23^ warned that over 40% of the region's biodiversity may vanish by 2100. Understanding the impact of interventions in this region, like protection status or carbon payments on retaining forest cover, is critical for improving conservation responses with finite resources and is a requirement for satisfying Monitoring, Evaluation and Reporting (MER) program requirements to access REDD+ and other funding streams (e.g., the Global Environmental Facility and the World Bank). Stronger forest protection and conservation efforts are needed across Southeast Asia’s existing protected areas to avert the future trajectories of forest cover and forest carbon loss estimated by 2050^24^.

Here, we evaluate the effectiveness of Southeast Asian protected areas at reducing forest cover loss and associated carbon loss, while controlling for spatial variation in deforestation pressure. We also explore whether the process of conducting management effectiveness reporting is correlated to improved protected area performance, and whether performance can be predicted by levels of management resourcing. Protection from logging, expansion of monoculture plantations and other threats is dependent on site-level factors, such as adequate investment in management activities, as well as system-level factors, such as national governance, yet the level of protection afforded by it is complex. We wanted to understand how these factors inter-relate, and link to the level of avoided forest cover loss and carbon emissions. Our work has implications for the usefulness of the Management Effectiveness Tracking Tool (METT)^25^ (a score-card to assess the adequacy of management in protected areas based on the IUCN management effectiveness framework and used in over 3,000 sites across the globe)^26^, for predicting conservation impact and for two global environmental agreements. Parties to the Convention on Biological Diversity^27^ are now preparing and revising their post-2020 biodiversity decadal targets and parties to the UN Framework Convention on Climate Change^28^ revise their nationally determined contributions to reduce emissions under the Paris Climate Agreement every five years. Both conventions cover commitments to reduce deforestation and ecosystem service loss through the long-term protection of natural systems, and it is crucial to understand the contribution of protected areas towards achieving these targets.

## Results

### Effectiveness of protected areas for reducing forest cover loss and carbon emissions

**Figure 1:**
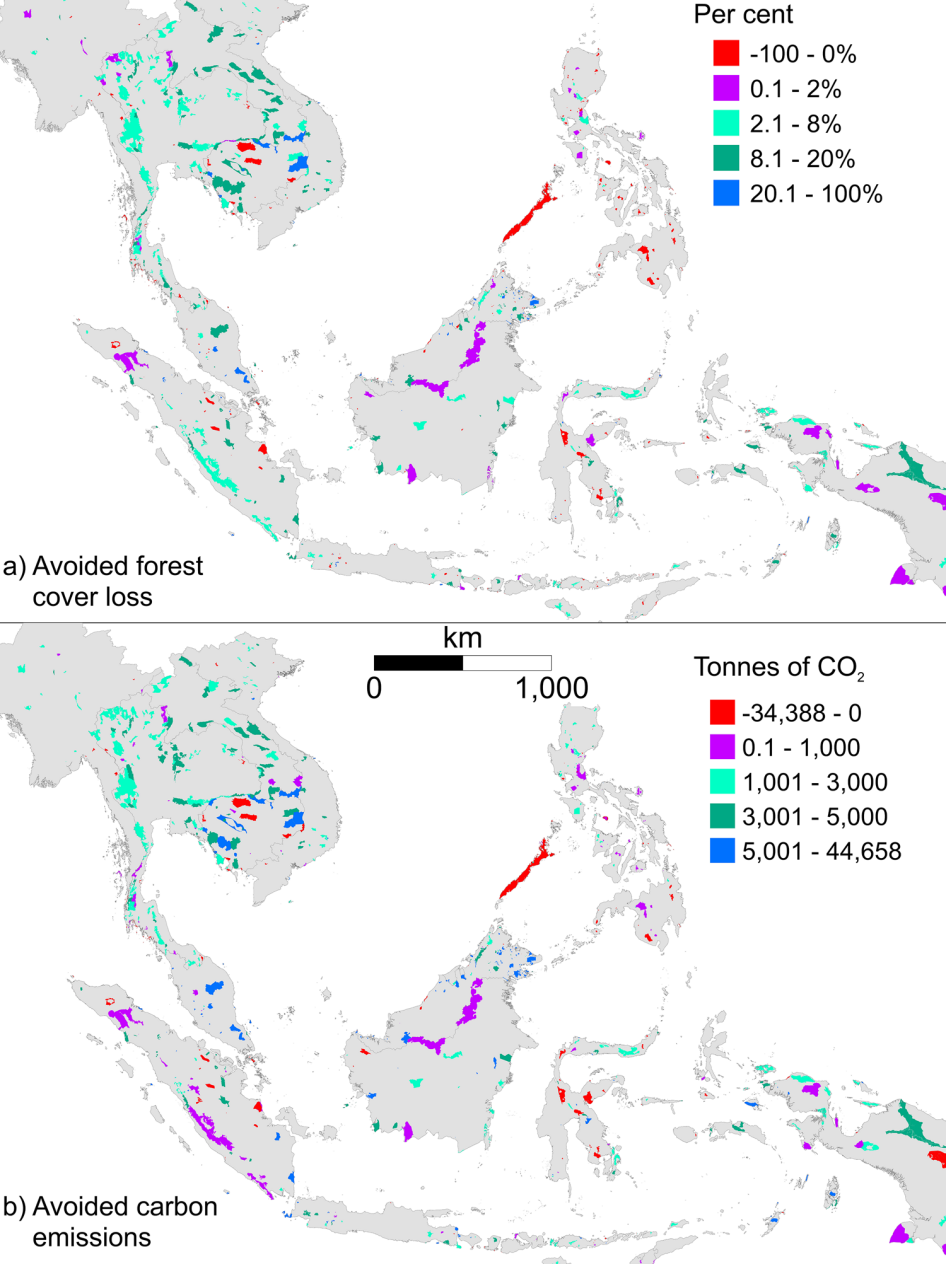
Estimated (**a**) forest cover loss and (**b**) carbon emissions (tonnes of CO_2_ per 1 km pixel) avoided in Southeast Asian protected areas from 2000–2018, aggregated to 1 km^2^ pixels. Negative values indicate that more forest cover was lost or more carbon was emitted inside the protected area than outside. The map was produced in ArcMap v10.5 (http://desktop.arcgis.com/en/arcmap/).

Our results show that protected areas significantly reduced the loss of forest cover with the overall rate of forest cover loss across the region being three times lower inside protected areas (n = 692) over the period 2000 to 2018 than in the matched unprotected landscape (*p* < 0.001; df = 76,679, t = − 49.55; Table 1). However, this overall effect masked important differences between countries, with Malaysia having the most effective protected area network, avoiding 14.57% of forest cover loss, followed by Cambodia (11.16%), Vietnam (10.27%), Laos (10.31%), Thailand (5.62%), Indonesia (3.93%), and Myanmar (2.28%). In the Philippines, protected areas lost 3 times more forest cover than unprotected areas, meaning not only was protection not effective, it in fact accelerated forest loss. Likewise, each country-level effect concealed differences between protected areas (Fig. 1).

**Table 1:** Estimates of forest cover loss and carbon emissions per 30 m pixel from 2000–2018, aggregated to 1 km^2^ pixels, from within and outside protected areas and avoided due to protection, before and after matching.

Country	Forest cover loss *inside*	Carbon emissions *inside*	Before matching				After matching			
			Forest cover loss *outside*	*Avoided* forest cover loss	Carbon emissions *outside*	*Avoided* carbon emissions	Forest cover loss *outside*	*Avoided* forest cover loss	Carbon emissions outside	*Avoided* carbon emissions
Cambodia	12.70%	4575	13.47%	0.77%	4492	− 123	23.86%	11.16%	8337	3762
Indonesia	2.75%	1494	14.60%	11.84%	5287	3777	6.68%	3.93%	3002	1509
Laos	3.33%	1989	12.42%	9.08%	4704	2790	13.65%	10.31%	5173	3184
Malaysia	3.15%	1475	25.34%	22.19%	9637	8151	17.72%	14.57%	8336	6862
Myanmar	0.27%	348	2.63%	2.39%	1705	1401	2.55%	2.28%	1426	1077
Philippines	2.26%	2023	0.67%	− 1.59%	1337	− 735	0.77%	− 1.49%	1577	− 446
Thailand	0.37%	642	0.47%	0.20%	1454	804	5.99%	5.62%	3109	2467
Vietnam	0.69%	1016	8.25%	7.56%	2613	1531	10.96%	10.27%	3542	2526
Average	3.19%	1.695	9.73%	6.55%	3904	2200	10.27%	7.08%	4313	2618

Measured on a 20% sample drawn from the whole region. Carbon emissions are tonnes of CO_2_ per km^2^.

Carbon emissions were likewise significantly lower in protected areas over the period 2000 to 2018 than in matched areas not under protection (*p* < 0.001; df = 76,679, t = − 67.87). Post-matching, the average rate of carbon emissions in protected areas was 2.5 times lower (1695t CO_2_ per km^2^) than the counterfactual (4313t CO_2_ per km^2^), resulting in a net treatment effect of 2618t CO_2_ per km^2^, equivalent to a saving of 145t CO_2_ per km^2^ per year.

**Figure 2:**
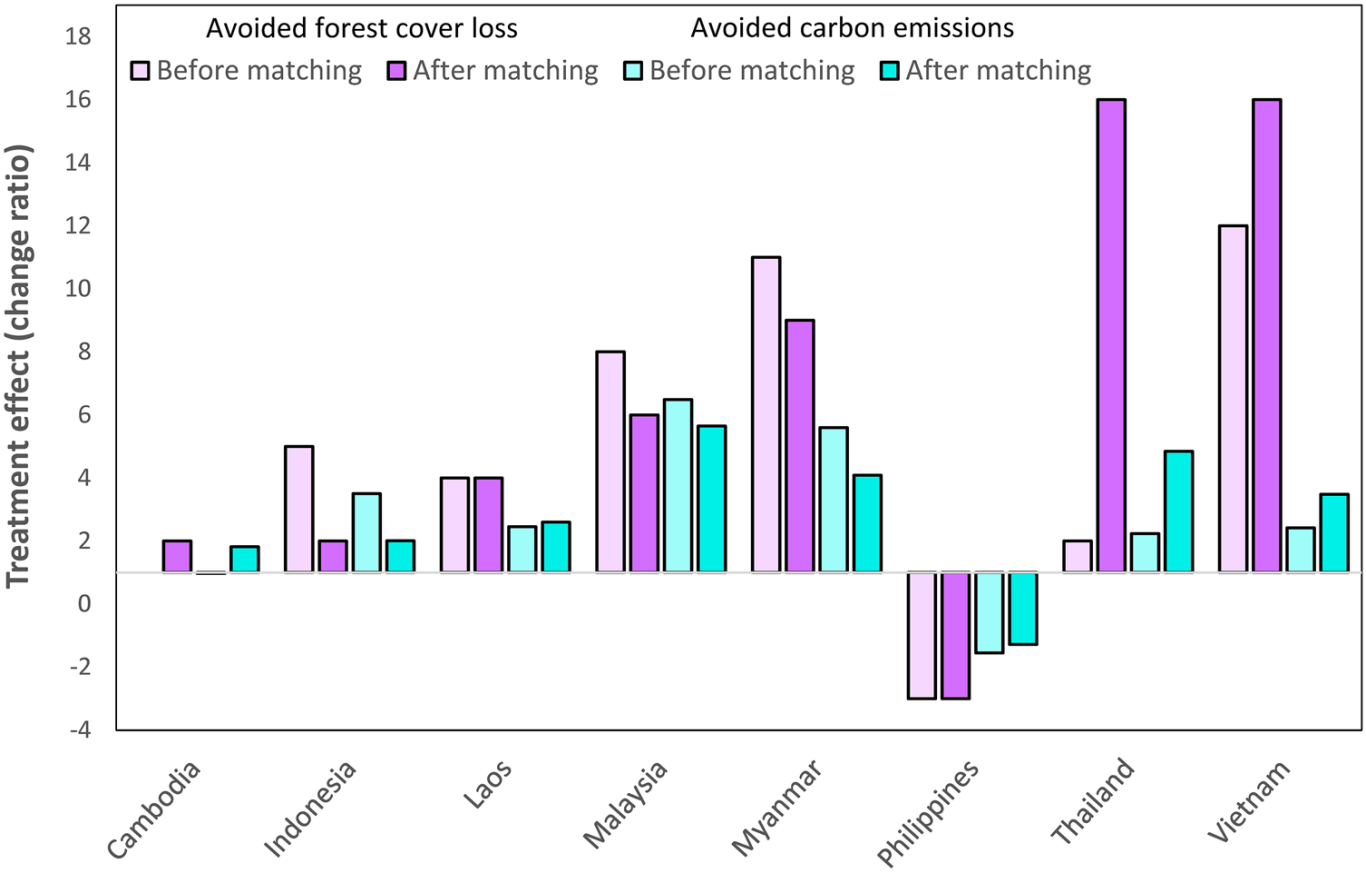
The treatment effect (change ratio) of protection before and after matching. Treatment effect of protection represents the level of forest cover loss and carbon emissions avoided before and after matching. Effect size is calculated by dividing the average forest cover loss rate per country in the counterfactual by the forest cover loss rate in the treatment area (protected area), or vice-versa when the latter is larger. The same formula is used for estimating the treatment effect for carbon emissions. Negative values indicate that more forest cover was lost, or more carbon was emitted, inside protected areas than outside.

The difference between the naïve (pre-matching) and counterfactual (post-matching) estimates of avoided forest cover loss and carbon emissions was small on average for the region (Table 1). However, for some countries, there were large changes in the magnitude of avoided forest cover and carbon emissions between pre- and post-matching and the direction of these changes varied inconsistently (Fig. 2). Estimates of avoided forest cover loss and carbon emissions decreased in 3 countries, increased in 3 countries and remained consistent in 2 countries. For example, Indonesian protected areas decreased from being 5 times more effective at reducing forest cover loss before matching, to only twice as effective than the counterfactual post-matching. Vietnam’s protected areas were 12 times more effective than the counterfactual prior to matching, yet after matching this factor increased to 16. Post-matching estimates of avoided forest cover loss and carbon emissions increased for Thailand, though the matching performance was poor, meaning the matching did not yield a large improvement in balancing the treatment and the counterfactual samples (S1).

### Impact of protected area monitoring and management reporting

**Figure 3:**
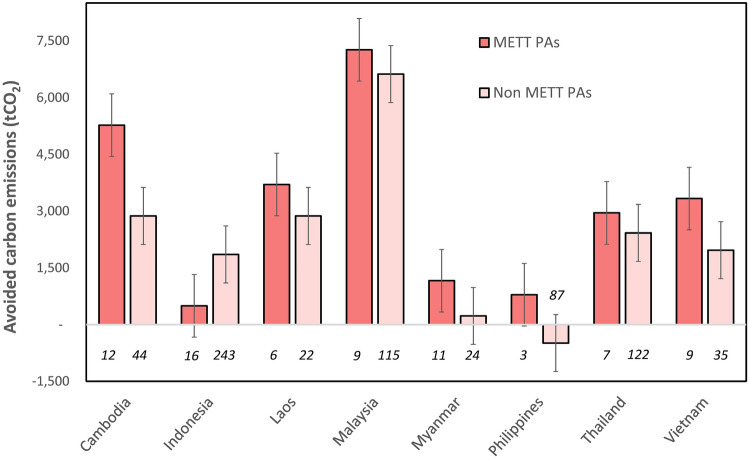
Rates of avoided forest cover loss for protected areas that had METT assessments, compared to those that had not. Units are rates of avoided forest cover loss for each country between 2000–2018 based on a 20% sample drawn from the whole region. The italicised numbers below the bars represent the number of protected areas within the group. Whiskers represent standard error bars.

**Figure 4:**
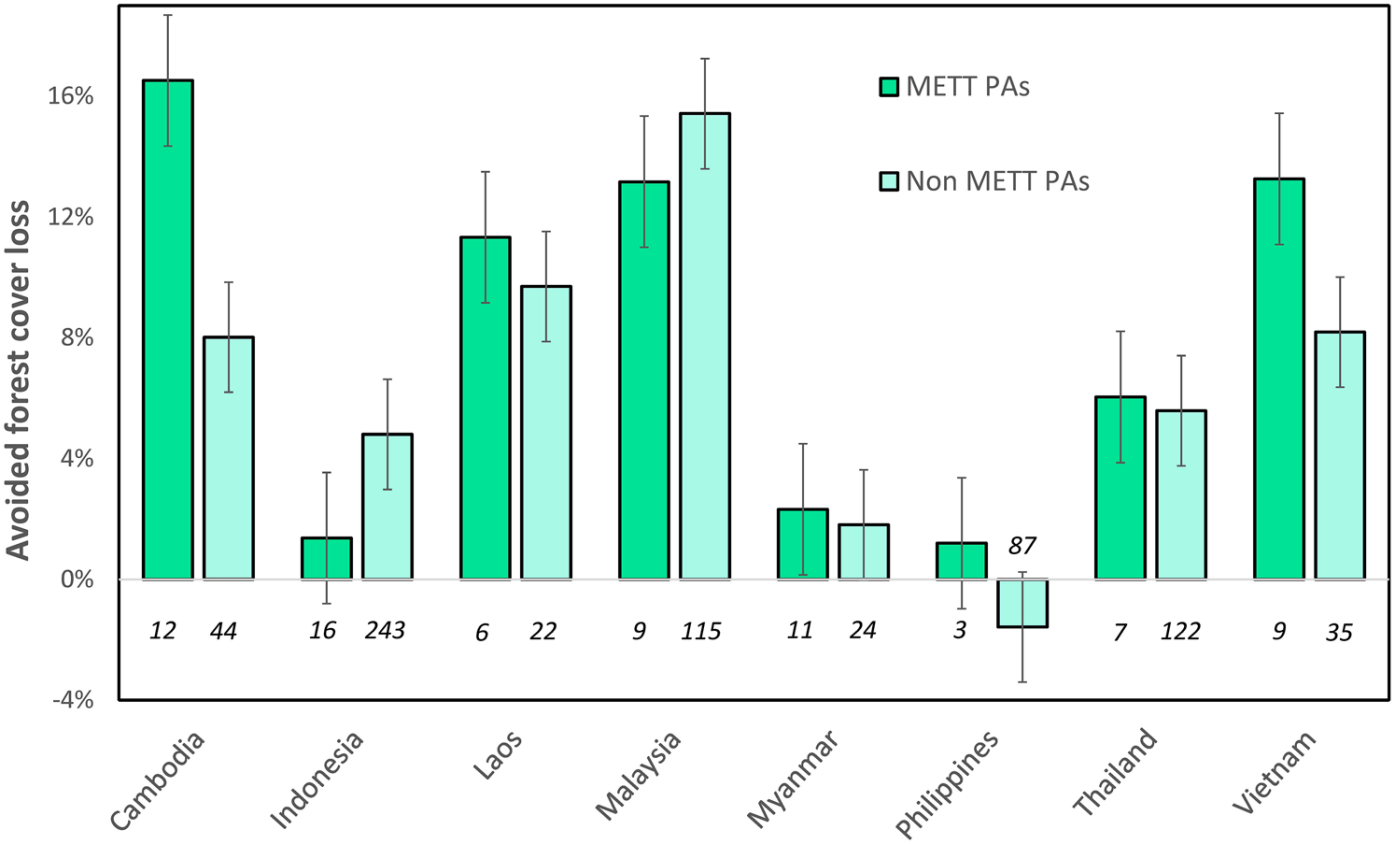
Avoided carbon emissions grouped by protected areas that had METT assessments, compared to those that had not. Units are tonnes of carbon emissions (tCO_2_) avoided per pixel (1 km^2^) between 2000–2018 based on a 20% sample drawn from the whole region. The italicised numbers below the bars represent the number of protected areas within the group. Whiskers represent standard error bars.

Our results show that protected areas with management effectiveness assessments had significantly reduced rates of forest cover loss and carbon emissions, with the overall rate of forest cover loss (*p* = 0.004) and carbon emissions (*p* = 0.016) significantly lower in protected areas that had completed METT assessments, compared to those that had not (Figs. 3 and 4). The average rate of forest cover loss avoided in METT protected areas was 9.67% (n = 73), compared to 8.10% in non-METT protected areas (n = 619). The average rate of carbon emissions avoided in protected areas that had METT assessments was 3500t CO_2_ per km^2^, compared to 2942t CO_2_ per km^2^ in the non-METT protected areas.

**Figure 5:**
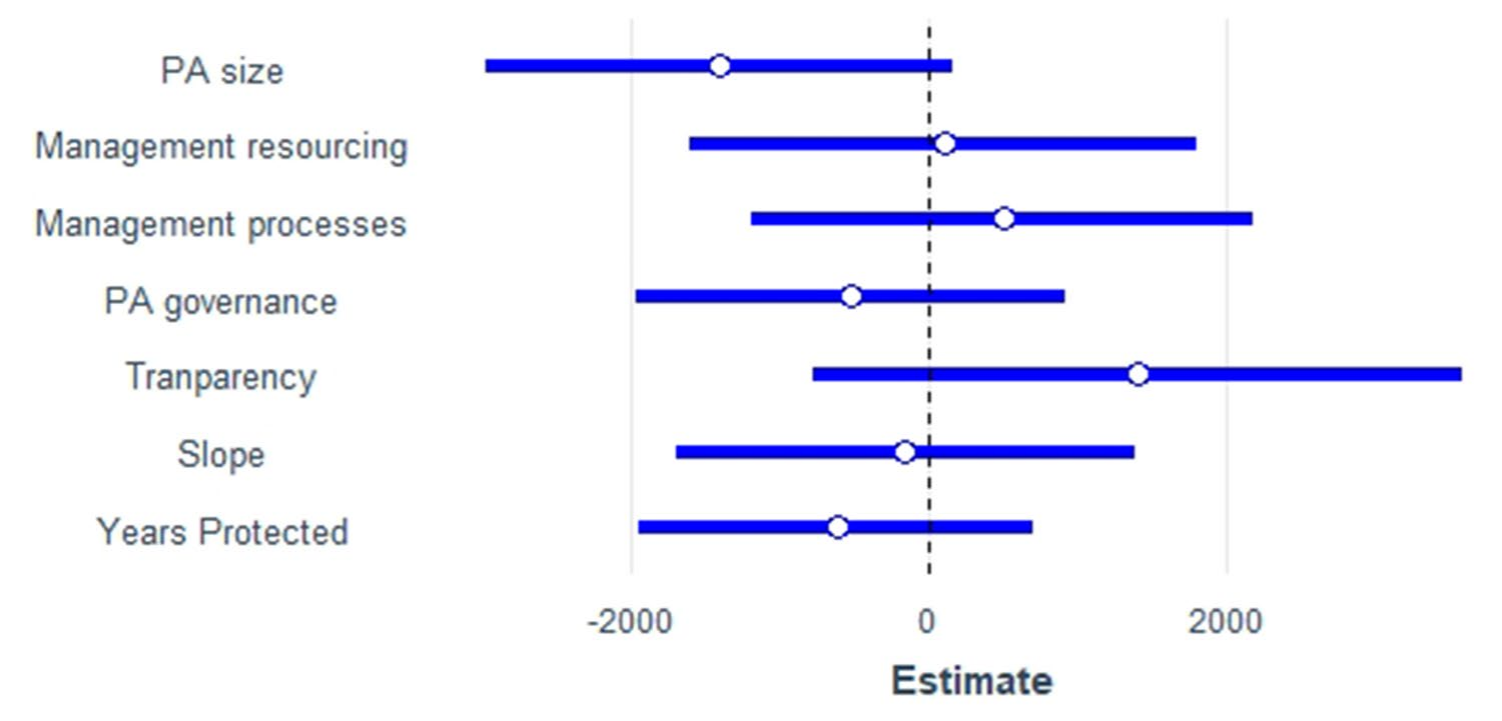
Regression coefficient estimates (scaled) of the model input variables in the best-fit linear mixed-effect models for predicting avoided carbon emissions in METT protected areas. Fit was assessed based on Akaike information criterion. Error bars are for a 95% confidence interval. Slope and protected area size were log-transformed to the power of 2 prior to fitting the model.

When exploring the individual components of the METT in more detail (Fig. 5), our most parsimonious model for predicting avoided forest cover loss did not detect a relationship between any of the management dimensions or contextual factors and avoided forest cover loss. However, for avoided carbon emissions, our most parsimonious model included protected area level management resourcing, design and planning, governance, national-level transparency, years protected, protected area size, human footprint index, oil palm suitability, and slope (Fig. 5). Country as a random effect explained 18% of the variance in the data.

## Discussion

Despite the intense level of human pressure Southeast Asian forests are facing^14^, we found the protected area network in the region lost 3 times less forest cover between 2000–2018 than the analogous unprotected landscape. This amounted to 2.5 times less carbon emissions in protected areas than the counterfactual. Our estimate is consistent with the findings from a global study^20^ that estimated Southeast Asian protected areas lost 3.3 times less intact forest between 2000–2013 than unprotected areas. These results also add to previous counterfactual matching studies from Sumatra and Borneo, that show protected areas resisted deforestation pressure over earlier periods^18,29^.

We found that statistical matching, which controlled for the non-random location of protected areas, had a variable effect on the direction and magnitude of our estimates of the protection impact. We found that post-matching estimates of avoided forest cover loss and carbon emissions decreased for Indonesia, Malaysia and Myanmar, remained stable for Laos and the Philippines, and increased for Thailand, Vietnam and Cambodia. Our finding contrasts with the consensus from the literature that the matched counterfactual approach consistently reduces the estimated impact of protection^17–19^. Joppa and Pfaff (2010) explain the reduced impact post-matching by the general bias in protected area placement towards places subject to low human pressure, which overestimates the protection effect. We did not find this pattern consistently across all countries. Bebber and Butt^30^ observed that controlling for residual placement bias was more important for regions with high forest cover. We suggest the causation mechanism underlying this pattern is for countries with high remaining forest cover, deforestation activities will be targeted towards unprotected forests over protected forests, whereas for countries with low remaining forest cover, protected areas must withstand escalating deforestation pressure due to lack of choice in available forest resources^31^. This theory offers a plausible explanation for Vietnam and the Philippines. Protected areas in the Philippines have the highest level of human pressure, yet protection appears to have the perverse impact of accelerating forest and forest carbon loss; a trend particularly evident on the island of Palawan where protected areas experienced more forest loss than expected^32^. We do not attempt to infer patterns of causation for the matched results for Thailand and Cambodia because the matching performance was poor (S1). To synthesize, we infer that these patterns may be driven by human pressure and proportion of remaining forest cover, though we recommend further in-depth exploration of the local socioeconomic conditions that may be driving this trend.

Our findings show that protection resulted in statistically significant lower levels of forest cover loss and carbon emissions, compared to the unprotected analogous landscape. Protected areas in the region have on average withstood escalating pressure relative to the analogous unprotected landscape in Cambodia, Indonesia, Laos, Malaysia, Myanmar, Thailand and Vietnam, but not in the Philippines. Regional deforestation trends in Southeast Asia are influenced largely by Indonesia, where over 50% of the region’s forests remain and where ~ 40% of protected areas are located. In 2017, the annual deforestation rate in Indonesia dropped by 60%, followed by another small decline in 2018. These positive results may be influenced by policy initiatives to slow deforestation and restore peatlands in Indonesia, such as Presidential Instruction No. 10/2011 suspending new concession licenses for logging and forest conversion in primary forest and peatlands, and the high volume of REDD+ projects^33,34^. Despite overall positive outcomes of protection, there are examples where protected areas were completely ineffective. For example, two Cambodian protected areas Roneam Daun Sam Wildlife Sanctuary and Snoul Wildlife Sanctuary lost 100% and 80% of forest cover respectively and both were degazetted by the Government of Cambodia shortly after. These examples highlight that without adequate resourcing, political backing or community support, protected areas can be degraded and degazetted for increased access to extractive activities^35^.

Protected areas with Management Effectiveness Tracking Tool (METT) assessments withstood significantly more forest cover loss and carbon emissions than non-METT protected areas. This finding highlights the value of investing in monitoring and evaluation programs to predict performance. We recommend further exploration to determine the causal link in this relationship. By benchmarking performance against best practice standards, protected area managers may be identifying key strengths and weaknesses and thereby improving future performance as a result of the monitoring process. We also note that as METT assessments are a requirement of GEF-funded projects, the protected areas attracting donor funding may be funded because they have better performance. Given our findings that management scores are positively linked to carbon savings, combined with growing evidence of positive correlations between management scores and biodiversity population trends^36–38^, the value gained by participating in protected area management effectiveness reporting programs is becoming clearer^39^. Yet only 11% of protected areas in Southeast Asia had completed METT surveys.

Our study is the first to find evidence that management effectiveness scores from the METT database can predict the level of carbon emissions avoided in protected areas. All other studies to our knowledge, that attempted to link avoided deforestation or reduced fire incidents to management scores at sites across South America and Madagascar, did not detect a clear pattern^40–45^. Further, most of these studies attempt to link management scores to deforestation or fire, not carbon emissions. Although we found avoided carbon emissions and forest cover loss were correlated (Supplementary Information), some key differences arose. Differences can be due to woody vegetation density, vegetation type, stem diameter at breast height and vegetation age^46^. This is particularly relevant given our increased knowledge of the contribution of tropical protected areas in achieving targets laid out in the Paris Agreement^30,47^. Tropical protected areas reduced ~ 29% of tropical deforestation emissions between 2000–2012, compared to expected deforestation^30^. Looking forward, the conservation, restoration, and improved management of tropical forests, mangroves, and peatlands could provide between 23–37% of the cost-effective mitigation solution required by 2030 to limit global warming to 2 °C^48,49^. To account for losses in natural forests that are replaced by monoculture plantations (e.g., rubber, oil palm), we recommend future research explore carbon trends to provide deeper insight into forest cover change dynamics.

One of the most important caveats to consider when interpreting these results is that we have not considered land tenure and land use categories in matched areas not under formal protection, as described in Schleicher et al.^50^. Land that aligns with the IUCN definition of a protected area and contained in the World Database of Protected Areas (WDPA), does not capture all land with conservation value. Community-managed forests are a growing governance arrangement in developing countries^51^ and have reduced rates of deforestation in Indonesia^52^. Separating the different types of land use in the counterfactual area not under protection would provide further insight into what is occurring outside the protected area estate. However, other effective area-based conservation measures (OECMs) are not listed in the WDPA, unless they are declared. Similarly, we grouped different types of protected areas into a singular treatment group, when in reality, there are varying levels of human perturbations allowed under different governance regimes^53^. Other studies have found that protected area strictness does not consistently improve environmental and social outcomes^12,54^. Future research assessing how protected area management and governance dimensions relate to social impacts would be highly valuable to understand their multi-dimensional impacts. Evidence exists of positive conservation and socioeconomic outcomes from protected areas^55^, particularly those that implement collaborative management regimes, where local people were empowered and maintained cultural and livelihood benefits^56^; yet no studies to our knowledge have linked management scores with social impacts.

In conclusion, by accounting for spatially-dynamic deforestation pressures, we find evidence that the Southeast Asian protected area network is conserving forests and forest carbon stocks, in addition to the biodiversity benefits previously reported^37^. Higher levels of management resourcing are associated with greater reductions in carbon emissions. The protected area network in the region therefore presents strong opportunities for scaled-up investment of conservation and REDD + finance. Stronger forest protection and conservation efforts are needed in Southeast Asia’s existing protected areas to avert projected trajectories of forest cover and forest carbon loss estimated by 2050^24^. However, protection requires funding to implement and less than 3% of all climate mitigation funding went to forest-based mitigation strategies^57,58^ One step towards mobilizing greater financial support is demonstrating strong links between management resourcing and conservation impacts. Given the low level of finance being directed at forest-based climate mitigation solutions, combined with the sizable contribution of tropical protected areas in achieving the Paris Agreement targets^30,48,59^, we recommend that financiers enhance stimulus for protection to more effectively store and sequester carbon. Our study shows that management effectiveness frameworks, such as the METT, can play a guiding role in predicting protection outcomes.

## Materials and methods

We analysed a total area of 747,714 protected and unprotected pixels, covering eight countries: Cambodia, Indonesia, Laos, Malaysia, Myanmar, the Philippines, Thailand, and Vietnam. Timor-Leste, Brunei, and Singapore were excluded from the analysis because there were insufficient pixels in these countries to do matching. Full details of our data sources and preparation are provided in Supplementary Information with a summary included below. All the data we used is freely available. All spatial analysis was performed in ArcMap v10.5 (ESRI 2016) using Asia South Albers Equal Area Conic projection. Statistical modeling was performed in R v3.4.3 (R Development Core Team 2017).

### Avoided forest cover loss and carbon emissions

We used the Hansen tree cover loss maps that measure the annual change in tree cover between 2000 and 2018 and the tree canopy cover map for the year 2000 at a spatial resolution of ∼30 m across the terrestrial world (version 1.6; Hansen et al.^60^). We used the aggregate tool in ArcMap to downscale from the ~ 30 m pixels to ~ 1 km resolution (Supplementary Information). Tree cover loss includes either outright deforestation or temporary disturbance (e.g. due to forestry, selective logging, shifting cultivation or wildfires^61^). To estimate forest cover loss between 2000 and 2018, we created a binary forest cover map for the year 2000, where forest was categorized as having tree cover of greater than or equal to 40% canopy cover and “loss” was restricted to only those pixels that were classified as “forest” in 2000. We identified this threshold by running comparisons of the total forest area resulting from a range of thresholds and comparing it against the total forest area in the Global Forest Watch platform (www.globalforestwatch.org). The rate of forest cover loss in each protected area was calculated by aggregating the total number of pixels that had lost forest cover over the 18-year period and dividing this total by the number of pixels sampled. For carbon emissions, we used the CO_2_ emissions from aboveground woody biomass loss between 2000 and 2018 at a resolution of ∼30 m across the tropics^62^. In this study, “carbon emissions” refers to emissions “committed” at the time of disturbance or clearing, noting there may be a time lag until they are “realized”. Again, we used the aggregate tool in ArcMap to downscale from the ~ 30 m pixels to ~ 1 km resolution by summing all values. Carbon emissions per protected area were calculated in the same way as forest cover loss, resulting in an average carbon emission saving per pixel. Our carbon emission estimates do not include emissions from peat.

We measured the treatment effect in terms of deforestation for each country, by dividing the average forest cover loss rate in the counterfactual by the forest cover loss rate in the treatment area (protected area), or vice-versa when the latter is larger. The same method was used to calculate the treatment effect in terms of carbon emissions. We used the unpaired Wilcoxon test to assess whether there were significant differences between treatment areas and the matched controls.

### Protected areas

We used a cleaned map of terrestrial protected areas that were established prior to the year 2000 from the March 2018 World Database on Protected Areas (WDPA; UNEP-WCMC 2018). To identify and remove overlaps between designations (e.g., international and national designation) and IUCN classifications, we created separate layers for each IUCN classification, erasing any overlap between the categories and retaining the strictest classification (Deguignet et al. 2017; see Supplementary Information). The terrestrial administrative boundaries used in the analysis are taken from the Database of Global Administrative Areas (GADM; www.gadm.org), version 2.8, November 2015.

### Definition of effectively managed protected areas

The International Union for the Conservation of Nature (IUCN) Green List framework evaluates protected area management effectiveness based on four components: (1) good governance, (2) sound design and planning, (3) effective management, and (4) successful conservation outcomes. It is a new global standard for assessing whether protected areas are achieving conservation outcomes through effective management and equitable governance (IUCN and WCPA 2017). However, because it is new, it has not yet been widely applied in protected area evaluations. The Management Effectiveness Tracking Tool METT;^25^ is the largest global source of information on protected area management effectiveness^63^.

We used METT assessments conducted between 2000 and 2014 as protected area management scores. We used the approach outlined in Graham et al.^37^ to select, exclude, and re-align METT survey responses to the four IUCN Green List Standard components based on congruence between objectives being measured by each indicator (also see Table S11 in Supplementary Information). We also excluded the conservation outcomes survey responses because we replaced this with an independent, quantitative measure of ‘conservation outcomes’, being ‘avoided deforestation’ and ‘avoided carbon emissions’, which is a more powerful indicator than a categorical score. The effective management component had more questions than any other category, therefore we split it into two sub-categories: management resourcing and management processes. We were left with the following four dimensions of management: (1) good governance, (2) sound design and planning, (3) management resourcing, and (4) management processes. Each assessment consists of 30 questions that are scored from 0 (inadequate or non-existing) to 3 (adequate or fully implemented). We selected the earliest possible assessment date because management factors should precede any resulting impact. Finally, we calculated an average score for all METT questions within these four groups.

### Statistical matching to assess protected area performance

We selected control sites to measure the impact of protection using the MatchIt package in R^64^. To account for the non-random distribution of protected areas, we included multiple predictive factors to balance the counterfactual and treatment samples. We based our approach for selecting predictive factors on analyses of deforestation in Southeast Asia^24,65^ and from global protected area impact studies using matching^13,14^. We selected anthropogenic drivers (human population density, presence of roads and railways, pressure to expand croplands and urban environments), suitability for land conversion (elevation, slope, and agricultural suitability), and biological aspects (peat characteristics and forest cover; see Table 2 for data sources and Supplementary Information for more details). To account for leakage of protected areas, we excluded buffers of 10 km around their boundaries. The buffer area is inappropriate as an independent control, because the area immediately outside a protection zone can be partially protected due to the close proximity to the restricted area^18^ or subject to higher deforestation when logging and other activities are displaced to the area immediately outside the protected area^66,67^. We randomly selected a sample covering 20% of the total study region to reduce spatial autocorrelation^68^. After removing missing values, we retained a final sample of 747,714 of 1 km^2^ pixels.

**Table 2:** Details, data sources and rationale for the variables selected for the statistical matching.

Type	Covariates	Rationale	Restrictions in matching	Data source
**Intervention variable**	Protected areas	Terrestrial protected areas established prior to 2000	Treatment	WDPA (www.protectedplanet.net)
**Control variables**				
**Political drivers**	Country	Political systems and environmental policies differ between countries, so matching must be contained within the same country	Restricted to country	GADM (https://gadm.org/data.html)
**Anthropogenic drivers**	Human Footprint Index: HFI	Human population density, roads, railways, pressure to expand croplands and urban environments negatively influence the location of PAs	Balance sample to treatment	HFP1993 (https://wcshumanfootprint.org/)
**Environmental drivers**	Elevation	Elevation accounted for in the selected location of PAs	Balance sample to treatment	Global Digital Surface Models (DSM), “ALOS World 3D-30 m” (AW3D30) (https://www.eorc.jaxa.jp/ALOS/en/aw3d30/index.htm)
	Slope	Slope accounted for in the selected location of PAs	Balance sample to treatment	Calculated from Elevation layer (as above)
	Agricultural suitability – oil palm, cassava, rice, maize	Highly suitable land for oil palm, cassava, rice or maize is more likely to be cleared and less likely to be protected	Balance sample to treatment	GAEZ class oil palm, cassava, wetland rice, maize (http://gaez.fao.org/Main.html#)
**Biological aspects**	Forest cover	Forests more likely to be protected	Balance sample to treatment	Hansen tree cover in 2000 v1.6 (https://earthenginepartners.appspot.com/science-2013-global-forest/download_v1.6.html)
	Peatlands	Peatlands have different biophysical characteristics and are more likely to be protected. Peninsular Malaysia and Indonesia	Balance sample to treatment	Peatlands, year 2000. Miettinen et al. 2012^69^ (https://crisp.nus.edu.sg/crisp_oview.html)
**Outcome variables**	Forest cover loss	A binary measure of forest cover loss for 2000–2018	Outcome	Hansen tree loss v1.6 2000–2018 (https://earthenginepartners.appspot.com/science-2013-global-forest/download_v1.6.html)
	Forest carbon loss	Biomass loss for 2000–2018	Outcome	Global Forest Watch Tree Biomass Loss (http://data.globalforestwatch.org/datasets/tree-biomass-loss)

**Figure 6:**
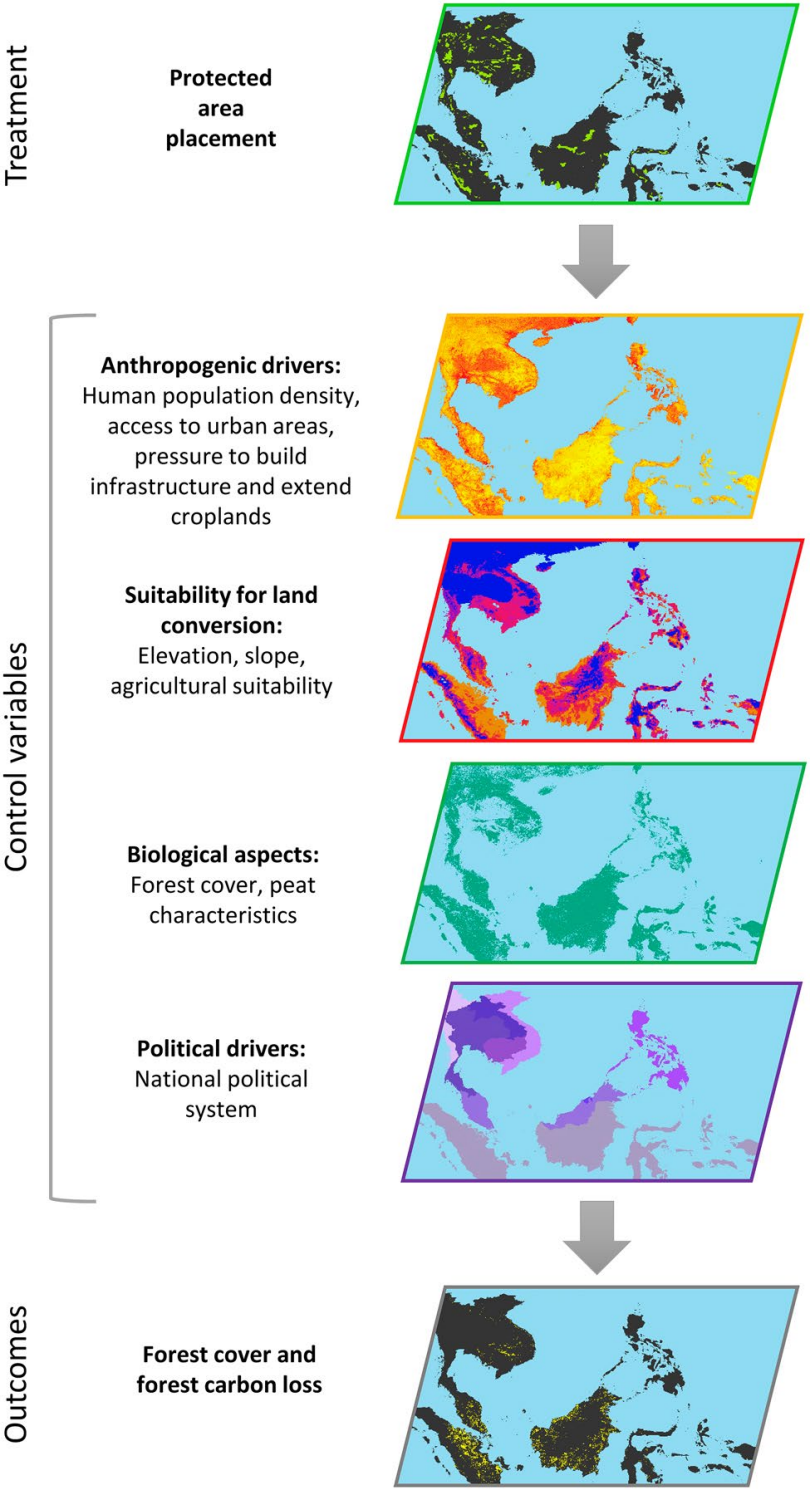
The broad statistical matching approach showing treatment, control and outcomes variables. To account for the non-random distribution of protected areas, we included multiple predictive (control) variables to create a statistically balanced counterfactual sample to evaluate protected area impact. We selected anthropogenic drivers, suitability for growing crops, and biological aspects. In each country, we selected a treatment and control sample, where the treatment is protection. Matching was repeated separately for each country. Once a suitable counterfactual sample was selected, we calculated the amount of forest cover loss and carbon emissions that were avoided due to protection.

We used Propensity Score Matching (PSM) to create a statistically balanced counterfactual sample to evaluate protected area impact for each country. Despite its limitations, PSM remains the most widely used matching approach^14,50,68^ and often performs comparatively better than other methods when handling a large numbers of covariates^70^. We tested coarsened exact matching (CEM) methods, however PSM performed better at finding paired matches for each pixel while maximising sample size. The unprotected pixels included in the matched analysis, represented the pixels most comparable to the protected pixels with respect to the matching variables within the same country (Fig. 6) using the nearest neighbour method, without replacement. We matched each treatment pixel to a unique control observation that had not been matched previously. We calculated avoided forest cover loss and carbon emissions at a pixel-by-pixel level, using one-for-one pairs.

### Approach for linking forest conservation to management

The forest cover loss rates during the period 2000–2018 and associated carbon emissions for each protected area was our unbiased measure of avoided forest cover loss and carbon emissions from protected areas. We built two predictive linear models that tested the direction and strength of the relationship between management and contextual factors, and the two dependent variables (1) avoided forest cover loss; and (2) avoided carbon emissions inside protected areas. Southeast Asian deforestation and degradation is largely driven by agriculture and logging, therefore anthropogenic drivers, accessibility and peat characteristics are the best determinants of the spatial patterns of deforestation^24^. Our independent variables included four management factors: (1) good governance, (2) sound design and planning, (3) management resourcing, and (4) management processes, as well as (5) years protected, (6) protected area size, (7) human pressure, (8) elevation, (9) slope, (10) agricultural suitability, (11) national government transparency, and (12) GDP (Supplementary Information). We selected the data that was nearest to the year 2000 for our contextual variables, prioritising older over more recent data, because management and contextual factors should precede any resulting impact. We performed correlation tests on all model variables. This resulted in removing GDP, elevation, and suitability for growing rice, cassava and maize due to high intercorreality. Slope and size were log-transformed to the power of two, to prevent outliers driving the results. The best-fit model was determined based on Akaike Information Criterion (AIC) of all possible configurations of predictor variables, using the MuMIn package^71^.

## Supplementary Information

Supplementary Information.

## Data Availability

The datasets used in this study are publicly available and may be provided by the corresponding author on reasonable request.
