# Supplementary material for: Southeast Asian protected areas are effective in conserving forest cover and forest carbon stocks compared to unprotected areas
Source: Sci Rep. 2021 Dec 9;11:23760. doi: 10.1038/s41598-021-03188-w (PMC8660836; doi:10.1038/s41598-021-03188-w)
Supplement: Supplementary file 1 — Supplementary Information. [file 41598_2021_3188_MOESM1_ESM.docx]

**Supplementary Information**

Southeast Asian protected areas are effective in conserving forest cover and forest carbon stocks compared to unprotected areas

Victoria Graham, Jonas Geldmann, Vanessa M. Adams, Pablo J Negret, Pablo Sinovas, Hsing-Chung Chang

**Supplementary methods: Data selection and preparation**

*Study region and sample size*

Our study region included eight countries (Cambodia, Indonesia, Laos, Malaysia, Myanmar, the Philippines, Thailand and Vietnam) across a total area of 4.5 million km^2^. All spatial layers were resampled to a 1km by 1km grid size to find a balance between the high-resolution data (forest cover loss; 30m) and the low-resolution data (e.g. agricultural suitability; 10km). We drew a 20% random sample from all pixels in the region for two reasons. First, to reduce spatial autocorrelation (Negret et al. 2020). Second, to work within computational power for the matching algorithm. After removing missing values, our final sample of 747,714 protected and unprotected pixels was retained. Our 20% sample was a good representation of the whole dataset (Figure S1).

*Protected areas*

Terrestrial protected areas from the World Database on Protected Areas (WDPA; UNEP-WCMC 2018) are comparable to locally-sourced maps of protected areas for Southeast Asia (Hughes 2018). Our analysis uses only polygons of protected areas and does not include point data that are lacking boundaries, since using buffered centroids from points for protected areas with unknown boundaries can result in large over- or underestimation of protection (Visconti *et al.* 2013). To identify and remove overlaps between designations (e.g. international and national designation) and IUCN classifications, we created separate layers for each IUCN classification, erasing any overlap between the categories and retaining the strictest classification (Deguignet *et al.* 2017). We did this by creating separate layers for each IUCN classification, and erasing any overlap between the categories: ia, ib, ii, iii, iv, v, vi, “not reported”, and “not applicable”. Then these 9 layers were merged to form a final boundary layer with no overlaps. For the biodiversity and forest cover change models, we apportioned the protected area attributes to the country with the majority coverage. For example; Thungyai Naresuan Wildlife Sanctuary cover both Thailand (3688 km^2^) and Myanmar (6km^2^), therefore we related this protected area to all Thailand country-level statistics.

*Management Effectiveness*

The IUCN Green List Standard evaluates protected area management effectiveness based on four components: (1) good governance, (2) sound design and planning, (3) effective management, and (4) successful conservation outcomes. It is a new global standard for assessing whether protected areas are achieving conservation outcomes through effective management and equitable governance (IUCN and WCPA 2017). However, because it is new, it has not yet been widely applied in protected area evaluations. The Management Effectiveness Tracking Tool METT; ^1^ is the largest global source of information on protected area management effectiveness ^2^.

We used METT assessments conducted between 2000 and 2014 as protected area management scores. We used the approach outlined in Graham et al. ^3^ to select, exclude and re-align survey responses to our predictors of interest. We selected the earliest possible date because management factors should precede any resulting impact. Each assessment consists of 30 questions that are scored from 0 (inadequate or non-existing) to 3 (adequate or fully implemented). We applied exclusion criteria to remove METT questions that were not directly linked to deforestation in the short-term, addressed the same criteria as another question, had no variation in scores, or used a different scoring method. We tested for collinearity between responses by performing Spearman rank correlations. This led to the exclusion of 7 covariates. We also excluded the METT question related to conservation outcomes because we replaced this with an independent measure of ‘conservation outcomes’, being avoided deforestation and carbon emissions. We were left with the following four dimensions of management: (1) good governance, (2) sound design and planning, (3) management resourcing, and (4) management processes ^4^. Finally, we calculated an average score for all METT questions within these four groups.

*Deforestation and carbon emissions data*

We used the Hansen lossyear and tree cover datasets to measure changes in forest cover between 2000 and 2018 (v1.6; Hansen et al. 2013). Tree cover loss includes any stand-replacement disturbance (degradation) or a change from forest to non-forest estate (deforestation). We used the sum method in the aggregate ArcGIS tool to downscale the data from the Hansen data (~30m spatial res) to ~1km resolution. To create a binary map of forest cover for years 2000 and 2018, we categorized forest as canopy cover greater than or equal to 40%. We identified this threshold by running comparisons of the total forest loss resulting from a range of thresholds (30%, 40%, 50%) and comparing it against the total forest loss in the Global Forest Watch platform (www.globalforestwatch.org). Then we combined the annual forest cover loss maps for each year between 2000 and 2018 to sum total loss for the 18-year period. The spatial resolution of our analysis does not detect all small-scale deforestation events or land cover changes from natural to plantation forests^5^, which resulted in our absolute forest loss being underestimated for Myanmar, the Philippines and Thailand.

Emissions are “gross” rather than “net” estimates, meaning that carbon stock gains are not included. Emissions associated with other carbon pools, such as soil carbon, are not included in these files. Loss of biomass may be from logging, fire and not strictly deforestation. We aggregated the 30m carbon emissions for all pixels in a 1km pixel to convert the estimates to our selected pixel size for the analysis.

*Contextual factors*

We used this cleaned layer as a base map to estimate protected area attributes (age and size). The elevation map we used was the global Digital Surface Models (DSM), “ALOS World 3D-30m” (AW3D30), which estimate land terrain at 30 meter spatial resolution ^6^(JAXA 2018). Surface maps represent the ground and anything detected above ground level (e.g. trees, buildings). We calculated slope from elevation. We used a map of cultivation suitability modelled using the Food and Agricultural Organization’s Global Agro-Ecological Zone methodology for four of the most exported crops from Southeast Asia: cassava, oil palm, rice, maize (FAO 2012). Perceived national government transparency was sourced from Transparency International ^7^. Elevation, slope and protected area size were log-transformed to the power of two, to prevent outliers driving the results. All data was scaled (mean = 0; SD = 1).

*Matching performance*

We considered the statistical matching performance was acceptable for the following reasons. After matching, the absolute standardised mean differences between the treatment and control of the confounding variables were all <0.1 for all countries, except Thailand (Figure S1). The difference in treatment effect in the prematch and postmatch samples was small (Tables S2-S9). The matching did not exclude any treatment units due to inability to identify a suitable control (Table S10). Therefore, propensity score matching identified comparable treatment and control groups. Any remaining difference in deforestation and carbon emissions between treatment and control groups could be attributed to the key predictor variable of the protection status.

**Supplementary Figures and Tables**
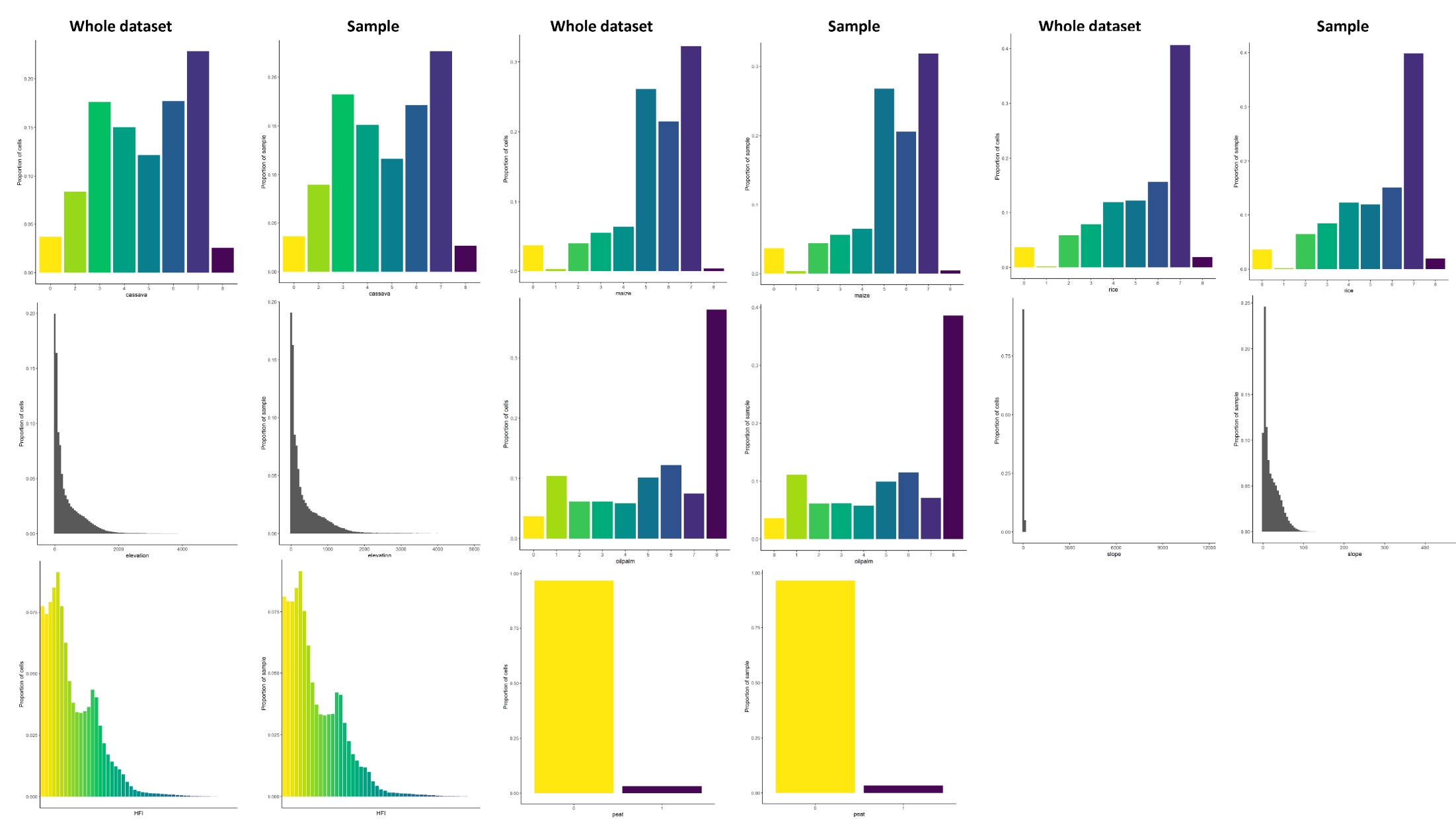


**Figure S1.** Distribution of covariates compared between the random sample of 20% of all pixels in the study region.


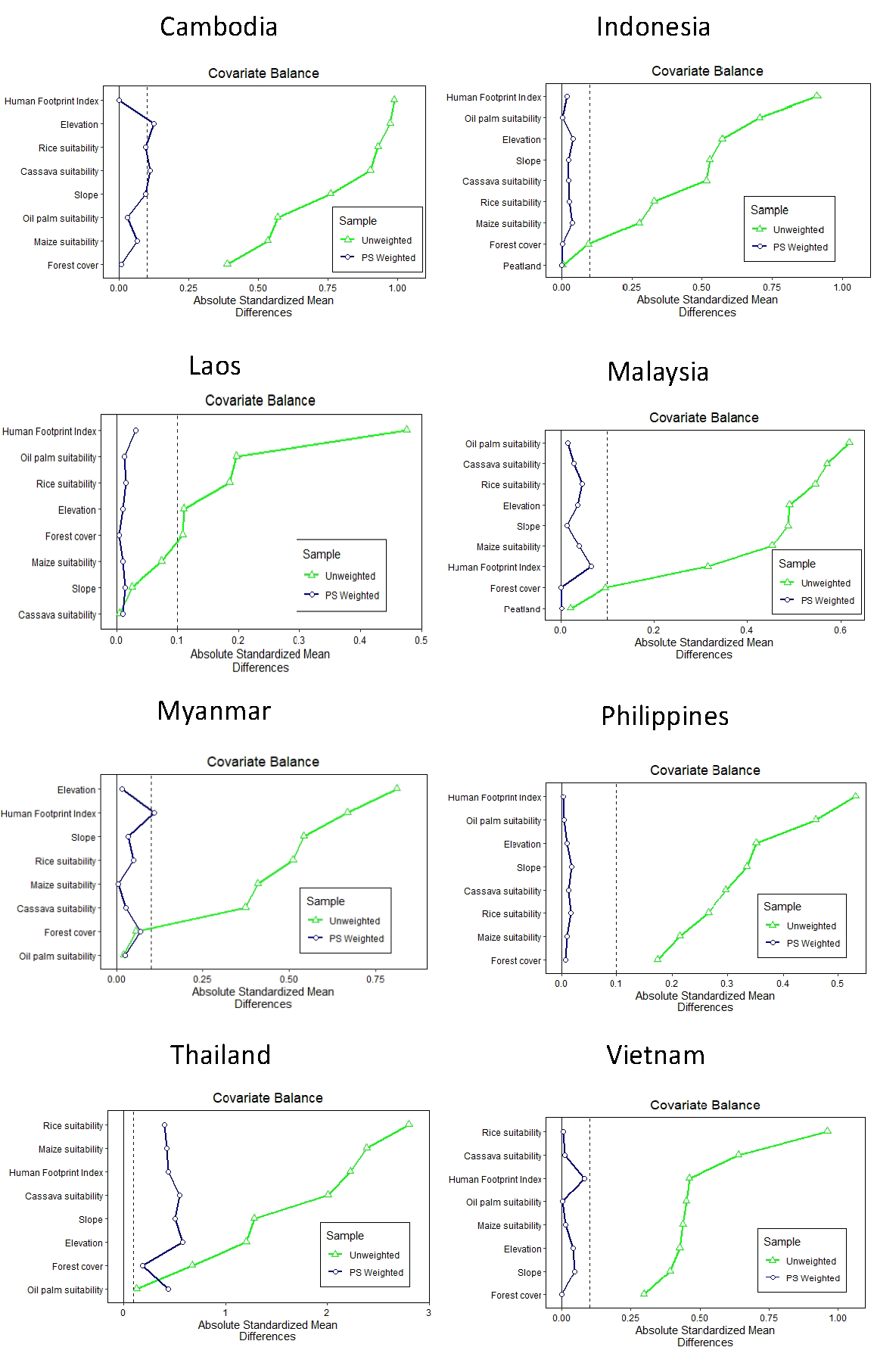


**Figure S2**. Love plots showing covariate balance for Cambodia, Indonesia, Laos, Malaysia, Myanmar, Philippines, Thailand and Vietnam before and after Propensity Score Matching.


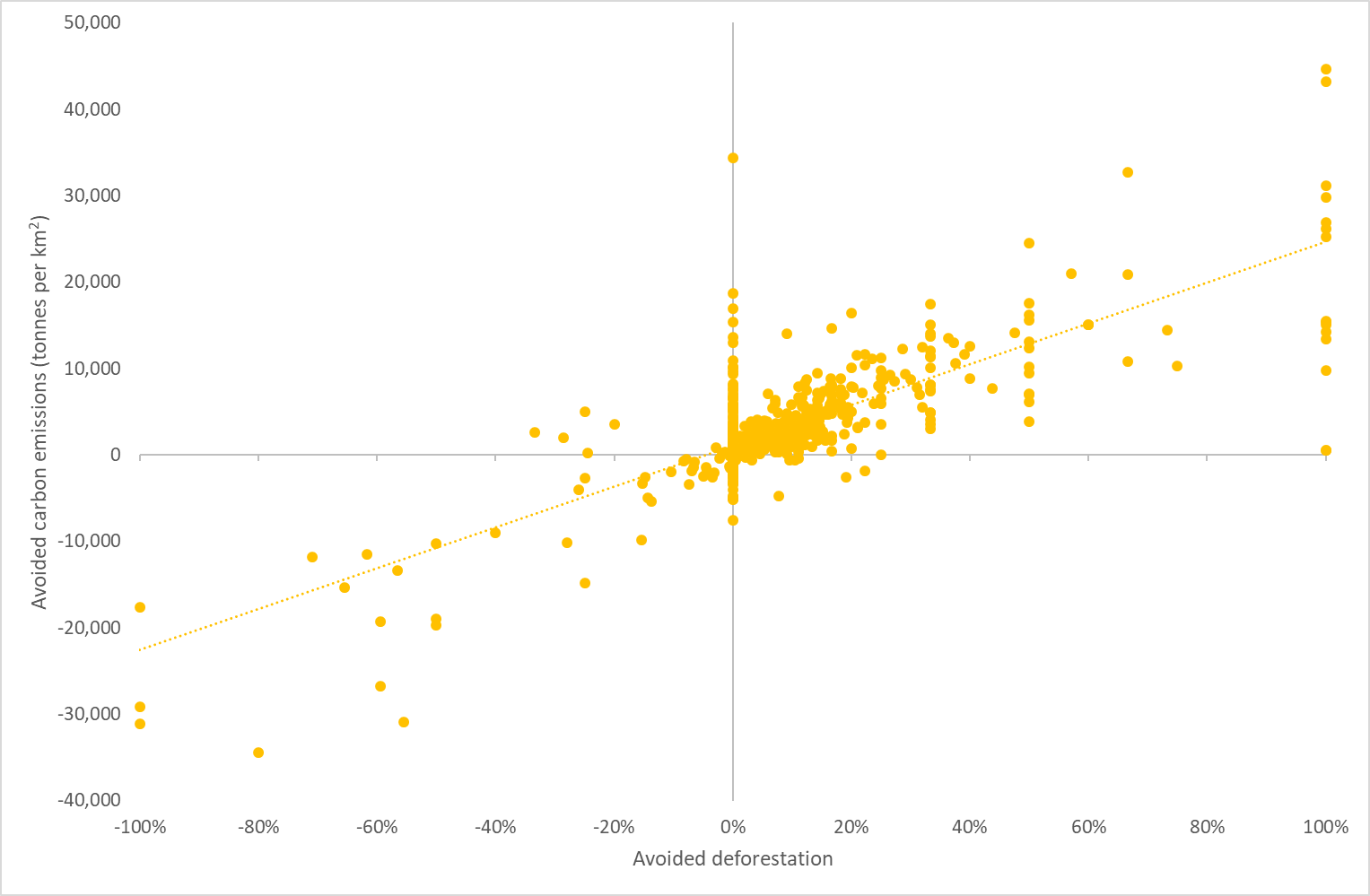


**Figure S3**. Relationship between our two impact metrics from protected areas: avoided deforestation (%) and avoided carbon emissions (tonnes of carbon per km^2^). Correlation = 0.79.


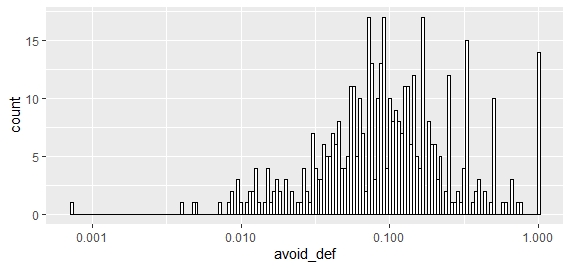


**Figure S4**. Frequency distribution of avoided deforestation for all protected areas in our sample.


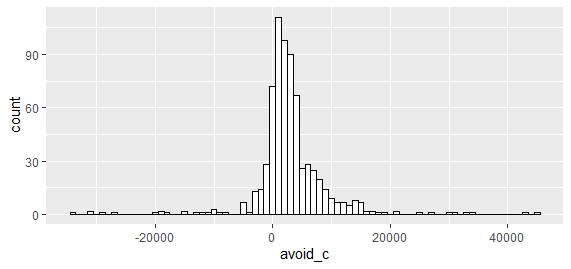


**Figure S5**. Frequency distribution of avoided carbon emissions for all protected areas in our sample.

**Table S1.** Regression coefficient estimates (scaled) of the input variables for the most parsimonious model based on Akaike information criterion for predicting avoided carbon emissions in protected areas. Error bars are for a 95% confidence interval. Slope and PA size were log-transformed to the power of 2 prior to fitting the model.

| **Model name** | **Dependent variable** | **Sample** | **AIC** | **R^2^** | **Positive associations** | **Negative associations** |
| --- | --- | --- | --- | --- | --- | --- |
| Model 1 (GLMM All) | Avoided carbon emissions | All PAs | 1506 | Multiple R^2^: 0.1804  Adjusted R^2^:0.05231 | government transparency, suitability for oil palm, slope | size, age, elevation, HFI |
| Model 2 (GLMM METT) | Avoided carbon emissions | METT subset | 1383 | R^2^m: 0.1088 R^2^c:0.2880 | METT 2, METT 3, government transparency, | HFI, oilpalm, size, age, slope, METT 4 |

**Table S2.** Overview of pre- and post-matching variables using **PSM** for all “nearest” matched variables for Cambodia

|  |  | Mean treatment | Mean Control | Std mean difference | eCDF Med * | eCDF Mean * | eCDF Max * | Percent balance improvement % |
| --- | --- | --- | --- | --- | --- | --- | --- | --- |
| before | cassava | 4.62 | 3.26 | 0.77 | 0.19 | 0.20 | 0.36 |  |
| after | cassava | 4.12 | 3.95 | 0.10 | 0.04 | 0.03 | 0.07 | 87.62 |
| before | maize | 5.00 | 4.21 | 0.45 | 0.05 | 0.13 | 0.36 |  |
| after | maize | 4.55 | 4.46 | 0.06 | 0.05 | 0.05 | 0.09 | 87.57 |
| before | oilpalm | 6.26 | 5.51 | 0.49 | 0.10 | 0.14 | 0.35 |  |
| after | oilpalm | 6.03 | 5.99 | 0.03 | 0.02 | 0.03 | 0.06 | 94.76 |
| before | peat | 0.00 | 0.00 | NaN | 0.00 | 0.00 | 0.00 |  |
| after | peat | 0.00 | 0.00 | NaN | 0.00 | 0.00 | 0.00 | 0.00 |
| before | rice | 5.30 | 3.76 | 0.86 | 0.24 | 0.21 | 0.38 |  |
| after | rice | 4.89 | 4.73 | 0.09 | 0.04 | 0.04 | 0.08 | 89.64 |
| before | slope | 13.22 | 5.11 | 0.59 | 0.28 | 0.25 | 0.36 |  |
| after | slope | 9.43 | 8.40 | 0.08 | 0.02 | 0.02 | 0.04 | 87.28 |
| before | forestcover | 0.74 | 0.35 | 0.89 | 0.19 | 0.19 | 0.39 |  |
| after | forestcover | 0.67 | 0.68 | -0.02 | 0.00 | 0.00 | 0.01 | 97.76 |
| before | elevation | 267.95 | 68.50 | 0.72 | 0.36 | 0.32 | 0.49 |  |
| after | elevation | 153.40 | 127.57 | 0.09 | 0.08 | 0.08 | 0.16 | 87.05 |
| before | HFI | 4.71 | 9.96 | -1.23 | 0.08 | 0.14 | 0.41 |  |
| after | HFI | 5.11 | 5.11 | 0.00 | 0.01 | 0.02 | 0.09 | 99.95 |

**Table S3.** Overview of pre- and post-matching variables using **PSM** for all “nearest” matched variables for Indonesia

|  |  | Mean treatment | Mean Control | Std mean difference | eCDF Med * | eCDF Mean * | eCDF Max * | Percent balance improvement % |
| --- | --- | --- | --- | --- | --- | --- | --- | --- |
| before | cassava | 5.39 | 4.34 | 0.52 | 0.11 | 0.13 | 0.28 |  |
| after | cassava | 5.39 | 5.45 | -0.03 | 0.01 | 0.01 | 0.05 | 94.90 |
| before | maize | 5.90 | 5.42 | 0.28 | 0.01 | 0.07 | 0.32 |  |
| after | maize | 5.90 | 5.96 | -0.04 | 0.01 | 0.01 | 0.03 | 85.56 |
| before | oilpalm | 5.75 | 3.81 | 0.70 | 0.27 | 0.21 | 0.38 |  |
| after | oilpalm | 5.75 | 5.73 | 0.00 | 0.02 | 0.02 | 0.05 | 99.46 |
| before | peat | 0.08 | 0.07 | 0.02 | 0.00 | 0.00 | 0.01 |  |
| after | peat | 0.08 | 0.08 | 0.00 | 0.00 | 0.00 | 0.00 | 98.89 |
| before | rice | 5.77 | 5.11 | 0.33 | 0.02 | 0.07 | 0.28 |  |
| after | rice | 5.77 | 5.83 | -0.03 | 0.01 | 0.01 | 0.05 | 91.71 |
| before | slope | 29.40 | 17.48 | 0.53 | 0.16 | 0.16 | 0.29 |  |
| after | slope | 29.40 | 29.94 | -0.02 | 0.01 | 0.01 | 0.03 | 95.46 |
| before | forestcover | 0.94 | 0.84 | 0.41 | 0.05 | 0.05 | 0.10 |  |
| after | forestcover | 0.94 | 0.95 | -0.02 | 0.00 | 0.00 | 0.00 | 95.29 |
| before | elevation | 664.86 | 288.72 | 0.57 | 0.21 | 0.19 | 0.33 |  |
| after | elevation | 664.86 | 692.98 | -0.04 | 0.02 | 0.02 | 0.05 | 92.52 |
| before | HFI | 2.91 | 5.97 | -0.91 | 0.02 | 0.06 | 0.22 |  |
| after | HFI | 2.91 | 2.85 | 0.02 | 0.00 | 0.01 | 0.04 | 97.93 |

**Table S4.** Overview of pre- and post-matching variables using **PSM** for all “nearest” matched variables for Laos

|  |  | Mean treatment | Mean Control | Std mean difference | eCDF Med * | eCDF Mean * | eCDF Max * | Percent balance improvement % |
| --- | --- | --- | --- | --- | --- | --- | --- | --- |
| before | cassava | 6.27 | 6.26 | 0.01 | 0.02 | 0.03 | 0.09 |  |
| after | cassava | 6.27 | 6.25 | 0.01 | 0.00 | 0.01 | 0.02 | -111.60 |
| before | maize | 6.47 | 6.40 | 0.07 | 0.04 | 0.04 | 0.07 |  |
| after | maize | 6.47 | 6.46 | 0.01 | 0.01 | 0.01 | 0.03 | 86.09 |
| before | oilpalm | 7.37 | 7.55 | -0.20 | 0.01 | 0.04 | 0.10 |  |
| after | oilpalm | 7.37 | 7.36 | 0.01 | 0.00 | 0.01 | 0.01 | 93.23 |
| before | peat | 0.00 | 0.00 | NaN | 0.00 | 0.00 | 0.00 |  |
| after | peat | 0.00 | 0.00 | NaN | 0.00 | 0.00 | 0.00 | 0.00 |
| before | rice | 6.69 | 6.56 | 0.19 | 0.01 | 0.02 | 0.06 |  |
| after | rice | 6.69 | 6.68 | 0.01 | 0.00 | 0.01 | 0.03 | 92.10 |
| before | slope | 33.54 | 33.01 | 0.03 | 0.02 | 0.02 | 0.07 |  |
| after | slope | 33.54 | 33.83 | -0.01 | 0.01 | 0.02 | 0.04 | 46.24 |
| before | forestcover | 0.94 | 0.83 | 0.45 | 0.05 | 0.05 | 0.11 |  |
| after | forestcover | 0.94 | 0.93 | 0.02 | 0.00 | 0.00 | 0.00 | 96.20 |
| before | elevation | 668.01 | 709.72 | -0.11 | 0.04 | 0.04 | 0.06 |  |
| after | elevation | 668.01 | 664.29 | 0.01 | 0.01 | 0.01 | 0.03 | 91.07 |
| before | HFI | 4.35 | 5.60 | -0.48 | 0.00 | 0.03 | 0.17 |  |
| after | HFI | 4.35 | 4.44 | -0.03 | 0.00 | 0.01 | 0.12 | 93.46 |

**Table S5.** Overview of pre- and post-matching variables using **PSM** for all “nearest” matched variables for Malaysia

|  |  | Mean treatment | Mean Control | Std mean difference | eCDF Med * | eCDF Mean * | eCDF Max * | Percent balance improvement % |
| --- | --- | --- | --- | --- | --- | --- | --- | --- |
| before | cassava | 5.82 | 4.98 | 0.57 | 0.08 | 0.10 | 0.26 |  |
| after | cassava | 5.82 | 5.86 | -0.03 | 0.01 | 0.01 | 0.03 | 95.19 |
| before | maize | 6.40 | 5.91 | 0.45 | 0.01 | 0.09 | 0.25 |  |
| after | maize | 6.40 | 6.44 | -0.04 | 0.01 | 0.02 | 0.05 | 91.45 |
| before | oilpalm | 5.54 | 4.24 | 0.62 | 0.17 | 0.14 | 0.27 |  |
| after | oilpalm | 5.54 | 5.57 | -0.01 | 0.01 | 0.01 | 0.03 | 97.64 |
| before | peat | 0.02 | 0.04 | -0.14 | 0.01 | 0.01 | 0.02 |  |
| after | peat | 0.02 | 0.02 | 0.01 | 0.00 | 0.00 | 0.00 | 91.79 |
| before | rice | 6.41 | 5.75 | 0.55 | 0.03 | 0.08 | 0.23 |  |
| after | rice | 6.41 | 6.47 | -0.05 | 0.00 | 0.01 | 0.04 | 91.72 |
| before | slope | 30.51 | 21.66 | 0.49 | 0.16 | 0.15 | 0.24 |  |
| after | slope | 30.51 | 30.74 | -0.01 | 0.01 | 0.01 | 0.03 | 97.38 |
| before | forestcover | 0.99 | 0.89 | 0.90 | 0.05 | 0.05 | 0.10 |  |
| after | forestcover | 0.99 | 0.99 | -0.01 | 0.00 | 0.00 | 0.00 | 98.98 |
| before | elevation | 467.60 | 255.69 | 0.49 | 0.20 | 0.19 | 0.33 |  |
| after | elevation | 467.60 | 451.94 | 0.04 | 0.02 | 0.03 | 0.08 | 92.61 |
| before | HFI | 5.11 | 6.49 | -0.32 | 0.02 | 0.03 | 0.10 |  |
| after | HFI | 5.11 | 4.83 | 0.06 | 0.00 | 0.01 | 0.11 | 79.59 |

**Table S6.** Overview of pre- and post-matching variables using **PSM** for all “nearest” matched variables for Myanmar

|  |  | Mean treatment | Mean Control | Std mean difference | eCDF Med * | eCDF Mean * | eCDF Max * | Percent balance improvement % |
| --- | --- | --- | --- | --- | --- | --- | --- | --- |
| before | cassava | 6.12 | 5.45 | 0.37 | 0.04 | 0.09 | 0.31 |  |
| after | cassava | 6.12 | 6.26 | -0.08 | 0.04 | 0.06 | 0.14 | 79.55 |
| before | maize | 6.15 | 5.45 | 0.42 | 0.06 | 0.09 | 0.24 |  |
| after | maize | 6.15 | 6.19 | -0.02 | 0.02 | 0.06 | 0.18 | 94.89 |
| before | oilpalm | 7.78 | 7.75 | 0.02 | 0.01 | 0.02 | 0.06 |  |
| after | oilpalm | 7.78 | 7.83 | -0.04 | 0.00 | 0.01 | 0.01 | -114.87 |
| before | peat | 0.00 | 0.00 | NaN | 0.00 | 0.00 | 0.00 |  |
| after | peat | 0.00 | 0.00 | NaN | 0.00 | 0.00 | 0.00 | 0.00 |
| before | rice | 6.67 | 5.83 | 0.55 | 0.08 | 0.10 | 0.29 |  |
| after | rice | 6.67 | 6.75 | -0.06 | 0.01 | 0.02 | 0.10 | 89.87 |
| before | slope | 41.23 | 25.13 | 0.45 | 0.12 | 0.13 | 0.23 |  |
| after | slope | 41.23 | 40.37 | 0.02 | 0.07 | 0.06 | 0.12 | 94.65 |
| before | forestcover | 0.69 | 0.63 | 0.12 | 0.03 | 0.03 | 0.06 |  |
| after | forestcover | 0.69 | 0.71 | -0.05 | 0.01 | 0.01 | 0.02 | 59.44 |
| before | elevation | 1459.48 | 564.76 | 0.62 | 0.18 | 0.18 | 0.30 |  |
| after | elevation | 1459.48 | 1394.04 | 0.05 | 0.07 | 0.06 | 0.13 | 92.69 |
| before | HFI | 4.54 | 7.65 | -0.81 | 0.01 | 0.07 | 0.32 |  |
| after | HFI | 4.54 | 4.58 | -0.01 | 0.00 | 0.01 | 0.08 | 98.45 |

**Table S7.** Overview of pre- and post-matching variables using **PSM** for all “nearest” matched variables for Philippines

|  |  | Mean treatment | Mean Control | Std mean difference | eCDF Med * | eCDF Mean * | eCDF Max * | Percent balance improvement % |
| --- | --- | --- | --- | --- | --- | --- | --- | --- |
| before | cassava | 5.07 | 4.46 | 0.30 | 0.07 | 0.08 | 0.16 |  |
| after | cassava | 5.07 | 5.10 | -0.01 | 0.00 | 0.00 | 0.01 | 95.55 |
| before | maize | 5.62 | 5.20 | 0.21 | 0.02 | 0.05 | 0.15 |  |
| after | maize | 5.62 | 5.64 | -0.01 | 0.00 | 0.01 | 0.03 | 95.15 |
| before | oilpalm | 5.47 | 4.37 | 0.46 | 0.11 | 0.12 | 0.29 |  |
| after | oilpalm | 5.47 | 5.48 | 0.00 | 0.01 | 0.02 | 0.06 | 99.12 |
| before | peat | 0.00 | 0.00 | NaN | 0.00 | 0.00 | 0.00 |  |
| after | peat | 0.00 | 0.00 | NaN | 0.00 | 0.00 | 0.00 | 0.00 |
| before | rice | 5.67 | 5.13 | 0.27 | 0.07 | 0.07 | 0.16 |  |
| after | rice | 5.67 | 5.70 | -0.02 | 0.01 | 0.01 | 0.05 | 93.59 |
| before | slope | 29.93 | 22.89 | 0.34 | 0.11 | 0.11 | 0.18 |  |
| after | slope | 29.93 | 30.32 | -0.02 | 0.01 | 0.01 | 0.03 | 94.45 |
| before | forestcover | 0.77 | 0.60 | 0.41 | 0.09 | 0.09 | 0.17 |  |
| after | forestcover | 0.77 | 0.78 | -0.02 | 0.00 | 0.00 | 0.01 | 95.77 |
| before | elevation | 447.12 | 295.07 | 0.35 | 0.12 | 0.11 | 0.19 |  |
| after | elevation | 447.12 | 442.61 | 0.01 | 0.02 | 0.01 | 0.04 | 97.04 |
| before | HFI | 9.46 | 11.83 | -0.53 | 0.02 | 0.05 | 0.20 |  |
| after | HFI | 9.46 | 9.47 | 0.00 | 0.01 | 0.01 | 0.04 | 99.31 |

**Table S8.** Overview of pre- and post-matching variables using **PSM** for all “nearest” matched variables for Thailand

|  |  | Mean treatment | Mean Control | Std mean difference | eCDF Med * | eCDF Mean * | eCDF Max * | Percent balance improvement % |
| --- | --- | --- | --- | --- | --- | --- | --- | --- |
| before | cassava | 6.13 | 3.87 | 2.01 | 0.24 | 0.28 | 0.65 |  |
| after | cassava | 6.13 | 5.51 | 0.55 | 0.03 | 0.08 | 0.22 | 72.72 |
| before | maize | 6.21 | 3.59 | 2.39 | 0.40 | 0.33 | 0.66 |  |
| after | maize | 6.21 | 5.74 | 0.42 | 0.01 | 0.06 | 0.22 | 82.27 |
| before | oilpalm | 7.35 | 7.22 | 0.13 | 0.03 | 0.05 | 0.15 |  |
| after | oilpalm | 7.35 | 6.92 | 0.44 | 0.03 | 0.05 | 0.15 | -236.00 |
| before | peat | 0.00 | 0.00 | NaN | 0.00 | 0.00 | 0.00 |  |
| after | peat | 0.00 | 0.00 | NaN | 0.00 | 0.00 | 0.00 | 0.00 |
| before | rice | 6.67 | 4.49 | 2.80 | 0.06 | 0.24 | 0.66 |  |
| after | rice | 6.67 | 6.36 | 0.40 | 0.01 | 0.04 | 0.17 | 85.72 |
| before | slope | 32.55 | 8.70 | 1.29 | 0.40 | 0.39 | 0.68 |  |
| after | slope | 32.55 | 23.14 | 0.51 | 0.17 | 0.15 | 0.24 | 60.57 |
| before | forestcover | 0.88 | 0.21 | 2.08 | 0.34 | 0.34 | 0.67 |  |
| after | forestcover | 0.88 | 0.69 | 0.57 | 0.09 | 0.09 | 0.19 | 72.40 |
| before | elevation | 582.98 | 195.24 | 1.21 | 0.42 | 0.39 | 0.65 |  |
| after | elevation | 582.98 | 398.35 | 0.58 | 0.21 | 0.18 | 0.27 | 52.38 |
| before | HFI | 6.16 | 13.90 | -2.23 | 0.04 | 0.16 | 0.60 |  |
| after | HFI | 6.16 | 7.70 | -0.44 | 0.01 | 0.05 | 0.24 | 80.20 |

**Table S9.** Overview of pre- and post-matching variables using **PSM** for all “nearest” matched variables for Vietnam

|  |  | Mean treatment | Mean Control | Std mean difference | eCDF Med * | eCDF Mean * | eCDF Max * | Percent balance improvement % |
| --- | --- | --- | --- | --- | --- | --- | --- | --- |
| before | cassava | 6.07 | 5.20 | 0.64 | 0.08 | 0.11 | 0.24 |  |
| after | cassava | 6.07 | 6.06 | 0.01 | 0.02 | 0.02 | 0.04 | 98.22 |
| before | maize | 6.31 | 5.86 | 0.44 | 0.03 | 0.06 | 0.22 |  |
| after | maize | 6.31 | 6.33 | -0.01 | 0.01 | 0.01 | 0.03 | 96.95 |
| before | oilpalm | 7.29 | 6.74 | 0.45 | 0.03 | 0.07 | 0.18 |  |
| after | oilpalm | 7.29 | 7.29 | 0.00 | 0.01 | 0.01 | 0.02 | 98.90 |
| before | peat | 0.00 | 0.00 | NaN | 0.00 | 0.00 | 0.00 |  |
| after | peat | 0.00 | 0.00 | NaN | 0.00 | 0.00 | 0.00 | 0.00 |
| before | rice | 6.57 | 5.48 | 0.96 | 0.11 | 0.12 | 0.27 |  |
| after | rice | 6.57 | 6.56 | 0.01 | 0.00 | 0.01 | 0.03 | 99.21 |
| before | slope | 33.90 | 25.25 | 0.39 | 0.13 | 0.13 | 0.24 |  |
| after | slope | 33.90 | 34.96 | -0.05 | 0.01 | 0.01 | 0.05 | 87.80 |
| before | forestcover | 0.78 | 0.48 | 0.72 | 0.15 | 0.15 | 0.30 |  |
| after | forestcover | 0.78 | 0.78 | 0.00 | 0.00 | 0.00 | 0.00 | 99.71 |
| before | elevation | 563.28 | 387.63 | 0.43 | 0.17 | 0.16 | 0.28 |  |
| after | elevation | 563.28 | 546.23 | 0.04 | 0.02 | 0.02 | 0.07 | 90.30 |
| before | HFI | 8.32 | 10.79 | -0.46 | 0.01 | 0.05 | 0.20 |  |
| after | HFI | 8.32 | 7.87 | 0.08 | 0.01 | 0.02 | 0.04 | 81.91 |

**Table S10.** Sample sizes for treatment and control before and after matching from a sample randomly selected covering 20% of the total study region.

|  |  | Control | Treatment |
| --- | --- | --- | --- |
| Cambodia |  |  |  |
|  | All | 19,741 | 8,283 |
|  | Matched | 8,283 | 8,283 |
|  | Unmatched | 11,458 | - |
| Indonesia |  |  |  |
|  | All | 293,499 | 33,858 |
|  | Matched | 33,858 | 33,858 |
|  | Unmatched | 259,641 | - |
| Laos |  |  |  |
|  | All | 31,133 | 6,749 |
|  | Matched | 6,749 | 6,749 |
|  | Unmatched | 24,384 | - |
| Malaysia |  |  |  |
|  | All | 47,476 | 4,063 |
|  | Matched | 4,063 | 4,063 |
|  | Unmatched | 43,413 | - |
| Myanmar |  |  |  |
|  | All | 112,203 | 2,068 |
|  | Matched | 2,068 | 2,068 |
|  | Unmatched | 110,135 | - |
| Philippines |  |  |  |
|  | All | 43,016 | 5,044 |
|  | Matched | 5,044 | 5,044 |
|  | Unmatched | 37,972 | - |
| Thailand |  |  |  |
|  | All | 63,010 | 16,255 |
|  | Matched | 16,255 | 16,255 |
|  | Unmatched | 46,755 | - |
| Vietnam |  |  |  |
|  | All | 56,895 | 1,159 |
|  | Matched | 1,159 | 1,159 |
|  | Unmatched | 55,736 | - |

**Table S11.** Alignment of two management effectiveness assessment frameworks

The 30 Management Effectiveness Tracking Tool (METT) questions aligned to the International Union for Conservation of Nature Green List of Protected and Conserved Areas Standard (IUCN Green List Standard) components. The left column is the IUCN Greenlist component and sub-component (underneath) and the middle column is the METT question/s that is aligned with that component. The right column identifies whether the METT question was included in our predictive model.

| IUCN Green List Component | METT Question | Included in model |
| --- | --- | --- |
| 1. Good Governance   *Demonstrate equitable and effective governance* | |  |
| 1.1. Guarantee legitimacy and voice | 7a. The planning process allows adequate opportunity for key stakeholders to influence the management plan. *Additional Point* | No |
|  | 22. Is there co-operation with adjacent land and water users? | No* |
|  | 23. Do indigenous and traditional peoples resident or regularly using the protected area have input to management decisions? | Yes |
|  | 24. Do local communities resident or near the protected area have input to management decisions? | Yes |
| 1.2. Achieve Transparency and Accountability | 1. Does the protected area have legal status (or in the case of private reserves is covered by a covenant or similar)? | No* |
| 1.3. Enable Governance Vitality and Capacity to Respond Adaptively | 7c. The results of monitoring, research and evaluation are routinely incorporated into planning. *Additional Point* | No |
| 1. Sound Design and Planning   *Clear, long-term conservation goals and objectives, based on a sound understanding of their natural, cultural and social-economic context* | | |
| 2.1. Identify and Understand Major Site Values | 9. Do you have enough information to manage the area? | Yes |
| 2.2. Design for Long-Term Conservation of Major Site Values | 5. Is the protected area the right size and shape to protect species, habitats, ecological processes and water catchments of key conservation concern? | Yes |
| 2.3. Understand Threats and Challenges to Major Site Values |  |  |
| 2.4. Understand the Social and Economic Context |  |  |
| 1. Effective Management | |  |
| 3.1. Develop and Implement a Long-Term Management Strategy | 4. Is management undertaken according to agreed objectives? | Yes |
|  | 7. Is there a management plan and is it being implemented? | Yes |
|  | 7b. There is an established schedule and process for periodic review and updating of the management plan *Additional point* | No |
|  | 8. Is there a regular work plan and is it being implemented? | Yes |
|  | 13. Are there enough people employed to manage the protected area? | Yes |
|  | 14. Are staff adequately trained to fulfil management objectives? | No* |
|  | 15. Is the current budget sufficient? | Yes |
|  | 16. Is the budget secure? | No |
|  | 17. Is the budget managed to meet critical management needs? | No* |
|  | 18. Is equipment sufficient for management needs? | Yes |
|  | 19. Is equipment adequately maintained? | No |
| 3.2. Manage Ecological  Condition | 11. Is there a programme of management- orientated survey and research work? | Yes |
|  | 12. Is active resource management being undertaken? | Yes |
| 3.3. Manage Within the Social and Economic Context of the Area | 21. Does land and water use planning recognise the protected area and aid the achievement of objectives? | No |
|  | 21a: Planning and management in the catchment or landscape containing the protected area incorporates provision for adequate environmental conditions (e.g. volume, quality and timing of water flow, air pollution levels etc) to sustain relevant habitats. *Additional Point* | No |
|  | 21b: Management of corridors linking the protected area provides for wildlife passage to key habitats outside the protected area (e.g. to allow migratory fish to travel between freshwater spawning sites and the sea, or to allow animal migration). *Additional Point* | No |
|  | 21c: Planning addresses ecosystem-specific needs and/or the needs of particular species of concern at an ecosystem scale (e.g. volume, quality and timing of freshwater flow to sustain particular species, fire management to maintain savannah habitats etc.) *Additional Point* | No |
|  | 24 a. Impact on communities: There is open communication and trust between local and/or indigenous people, stakeholders and protected area managers. *Additional Point* | No |
|  | 24b. Impact on communities: Programmes to enhance community welfare, while conserving protected area resources, are being implemented. *Additional Point* | No |
|  | 24c. Impact on communities: Local and/or indigenous people actively support the protected area. *Additional Point* | No |
| 3.4. Manage Threats | 2. Are appropriate regulations in place to control land use and activities (e.g. hunting)? | No* |
|  | 30b: Condition of values: Specific management programmes are being implemented to address threats to biodiversity, ecological and cultural values. *Additional Point* | No |
| 3.5. Effectively and Fairly Enforce Laws and Regulations | 3. Can staff (i.e. those with responsibility for managing the site) enforce protected area rules well enough? | Yes |
| 3.6. Manage Access, Resource Use and Visitation | 6. Is the boundary known and demarcated?  10. Are systems in place to control access/resource use in the protected area?  20. Education and awareness. Is there a planned education programme linked to the objectives and needs?  27. Are visitor facilities adequate?  28. Do commercial tour operators contribute to protected area management?  29. If fees (i.e. entry fees or fines) are applied, do they help protected area management? | Yes  No*  No  No  No  No |
| 3.7. Measure Success | 26. Are management activities monitored against performance?  30a: Condition of values: The assessment of the condition of values is based on research and/or monitoring. *Additional Point*  30c: Condition of values: Activities to maintain key biodiversity, ecological and cultural values are a routine part of park management. *Additional Point* | No*  No  No |
| 1. Conservation Outcomes   *Demonstrate successful long-term conservation of major natural values, and associated cultural and ecosystem service values; as well as the achievement of social and economic goals and objectives* | | |
| 4.1. Demonstrate Conservation of Major Natural Values | 30. What is the condition of the important values of the protected area as compared to when it was first designated? | No |
| 4.2. Demonstrate Conservation  of Ecosystem Services | 25. Is the protected area providing economic benefits to local communities, e.g. income, employment, payment for environmental services? | No |
| 4.3.Demonstrate Conservation of Cultural Values |  |  |

*Removed due to intercorreality

**Web References**

1 Stolton, S. *et al.* Reporting progress in protected areas a site level management effectiveness tracking tool. (Gland, Switzerland, 2007).

2 Coad, L. *et al.* Measuring impact of protected area management interventions: Current and future use of the global database of protected area management effectiveness. *Philosophical Transactions of the Royal Society of London B: Biological Sciences* **370** (2015).

3 Graham, V. *et al.* Management resourcing and government transparency are key drivers of biodiversity outcomes in southeast asian protected areas. *Biol. Conserv.* **253**, 108875, doi:<https://doi.org/10.1016/j.biocon.2020.108875> (2021).

4 Geldmann, J. *et al.* A global analysis of management capacity and ecological outcomes in terrestrial protected areas. *Conserv Lett* **11**, e12434 (2018).

5 Sexton, J. O. *et al.* Conservation policy and the measurement of forests. *Nature Clim Change* **6**, 192-196, doi:10.1038/nclimate2816

<http://www.nature.com/nclimate/journal/v6/n2/abs/nclimate2816.html#supplementary-information> (2016).

6 JAXA. (ed Japan Aerospace Exploration Agency) (2015).

7 International, T. *Corruption perceptions index*, <<www.transparency.org>> (2018).
